# Retrieval of 3D information in X-ray dark-field imaging with a large field of view

**DOI:** 10.1038/s41598-021-02960-2

**Published:** 2021-12-06

**Authors:** Jana Andrejewski, Fabio De Marco, Konstantin Willer, Wolfgang Noichl, Theresa Urban, Manuela Frank, Alex Gustschin, Pascal Meyer, Thomas Koehler, Franz Pfeiffer, Julia Herzen

**Affiliations:** 1 Chair of Biomedical Physics, Department of Physics, School of Natural Sciences, Technical University of Munich, 85748 Garching, Germany; 2 Munich Institute of Biomedical Engineering, Technical University of Munich, 85748 Garching, Germany; 3 Institute of Microstructure Technology, Karlsruhe Institute of Technology, 76344 Eggenstein-Leopoldshafen, Germany; 4 Philips GmbH Innovative Technologies, Research Laboratories, 22335 Hamburg, Germany; 5 Department of Diagnostic and Interventional Radiology, School of Medicine and Klinikum rechts der Isar, Technical University of Munich, 81675 München, Germany; 6 Institute for Advanced Study, Technical University of Munich, 85748 Garching, Germany

**Keywords:** Biomedical engineering, X-rays, Three-dimensional imaging, Radiography

## Abstract

X-ray dark-field imaging is a widely researched imaging technique, with many studies on samples of very different dimensions and at very different resolutions. However, retrieval of three-dimensional (3D) information for human thorax sized objects has not yet been demonstrated. We present a method, similar to classic tomography and tomosynthesis, to obtain 3D information in X-ray dark-field imaging. Here, the sample is moved through the divergent beam of a Talbot–Lau interferometer. Projections of features at different distances from the source seemingly move with different velocities over the detector, due to the cone beam geometry. The reconstruction of different focal planes exploits this effect. We imaged a chest phantom and were able to locate different features in the sample (e.g. the ribs, and two sample vials filled with water and air and placed in the phantom) to corresponding focal planes. Furthermore, we found that image quality and detectability of features is sufficient for image reconstruction with a dose of 68 μSv at an effective pixel size of $0.357 \times {0.357}\,\mathrm{mm}^{2}$. Therefore, we successfully demonstrated that the presented method is able to retrieve 3D information in X-ray dark-field imaging.

## Introduction

Shortly after the discovery of X-rays, attempts to extract three dimensional (3D) information from X-ray images were made and a number of techniques were developed to retrieve sectional images of the human body^1^. These early techniques, nowadays called ‘classic tomography’, used blurring of features outside a focal plane, by moving source and X-ray film during a single exposure, to enhance the detectability of features inside the focal plane^1^. However, in this method of body section imaging, features outside the focal plane are not completely removed from the images. This was omitted in later techniques by rotating source and detector around the patient. Image slices can then be reconstructed by computer-aided back projection. Hounsfield and Cormack developed the first medical computed tomography (CT) device in the 1970s^2,3^. Nowadays, CT is a widely used diagnostic tool. With the availability of digital flat panel detectors, body section imaging in form of tomosynthesis and limited angle tomography experienced a revival. Depending on the clinical questions, it has the potential of providing 3D information at lower dose and lower cost than CT. The principles of tomosynthesis are similar to those of classical tomography. The source is moved on linear or circular pathways around the object while the detector often remains stationary. However, in contrast to the early body section imaging techniques, multiple focal planes can be reconstructed from an acquisition consisting of several low-dose exposures. Thus, the dose of a tomosynthesis acquisition is often similar or slightly higher than for single radiographic images^4^. Especially breast tomosynthesis is a widely researched clinical application, which has also found its way into clinical practise^5,6^. Another tomographic approach is line trajectory X–ray tomography. Here, the source and detector remain stationary while the sample is moved in a linear trajectory while radiographs are recorded. After reconstruction, different focal planes can be obtained^7,8^.

These methods are based on the absorbing properties of a sample. However, also wave-optical effects such as refraction and small-angle scattering of X-rays occur at material interfaces. As these interactions with matter contain additional information of the sample, several methods were developed to retrieve phase shift and scattering information of objects^9–14^. One of these methods is grating-based X-ray imaging^15^. Here, three imaging modalities, namely attenuation, differential-phase-contrast, and dark-field signal, are obtained simultaneously. The dark-field signal is a measure of small angle scatter of X-rays induced by a sample^15–18^. A large number of studies of X-ray dark-field radiography and CT concerning small samples were performed^19–24^. Also, studies on X-ray phase-contrast and dark-field tomosynthesis were conducted, many using filtered back projection as a reconstruction method^25–28^. However, translating this method to large samples is not straightforward, especially as the fabrication of large gratings is difficult. Nevertheless, methods for X-ray dark-field radiography of large objects (in the vicinity of $30 \times {30}\,\mathrm{cm}^{2}$) were developed^29–34^. In this paper we present a method to obtain 3D X-ray dark-field information of large objects, which is similar to classic tomography, tomosynthesis and line trajectory X–ray tomography.

## Methods

### Imaging setup

**Figure 1:**
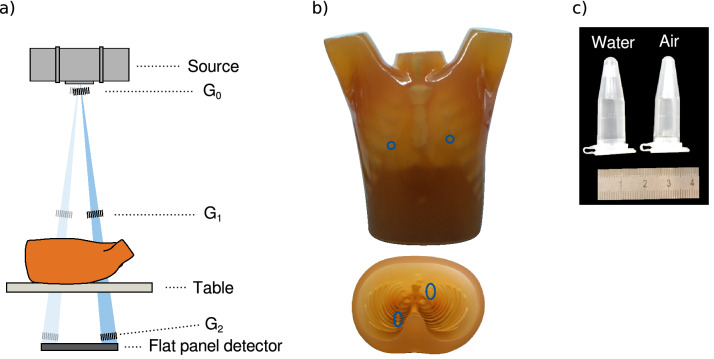
(**a**) Schematic of the setup. The gratings are mounted on an interferometer frame, which can rotate around the focal spot of the source. Two possible interferometer frame positions are shown. (**b**) Photograph of the imaged phantom. The coronal view in posterior direction on the top and the transverse view in superior direction is shown on the bottom. The position of the sample vials filled with water and air are indicated by the blue circles. (**c**) A photograph of the sample vials filled with water and air. Figure (**a**) adapted from Gromann et al. (cf. Ref^29^) according to ‘CC BY 4.0’ (https://creativecommons.org/licenses/by/4.0/).

The setup, depicted in Fig. 1a), is a three grating Talbot–Lau interferometer with identical inter-grating distances of 0.91 m and grating periods of 10 μm (symmetrical arrangement) . The two absorption gratings $G_0$ and $G_2$ are manufactured with the LIGA method^35^ and feature attenuating gold structures of a height of 200 μm. $G_2$ consists of eight single grating tiles with a size of 55 × ${75}\,\mathrm{mm}^{2}$ each, resulting in a total $G_2$ area of 440 × ${75}\,\mathrm{mm}^{2}$. The phase grating $G_1$ is a silicon grating manufactured with the DRIE method and a height of 60 μm. All gratings are mounted on a frame, which can rotate around the focal spot of the source. The setup is installed in a vertical arrangement with the detector at the floor level and the X-ray source placed vertically above it, with the source spot at a distance of ${199}\,\mathrm{cm}$. The sample is placed on a table, which is positioned ${35}\,\mathrm{cm}$ above the grating $G_2$. A motorised translation stage (LES 5, isel AG Germany, Dermbach, Germany) moves the table perpendicular to the grating lines. The imaging components of the setup are an actively-cooled medical X-ray source with a tungsten anode (MRC 0310 ROT GS, Philips Medical Systems, Hamburg, Germany), operated at 60 kV tube voltage and 600 mA tube current, and a flat-panel detector with an effective pixel size of 357 μm in the table plane (Pixium RF 4343, Trixell, Moirans, France).

### Data acquisition and processing

**Figure 2:**
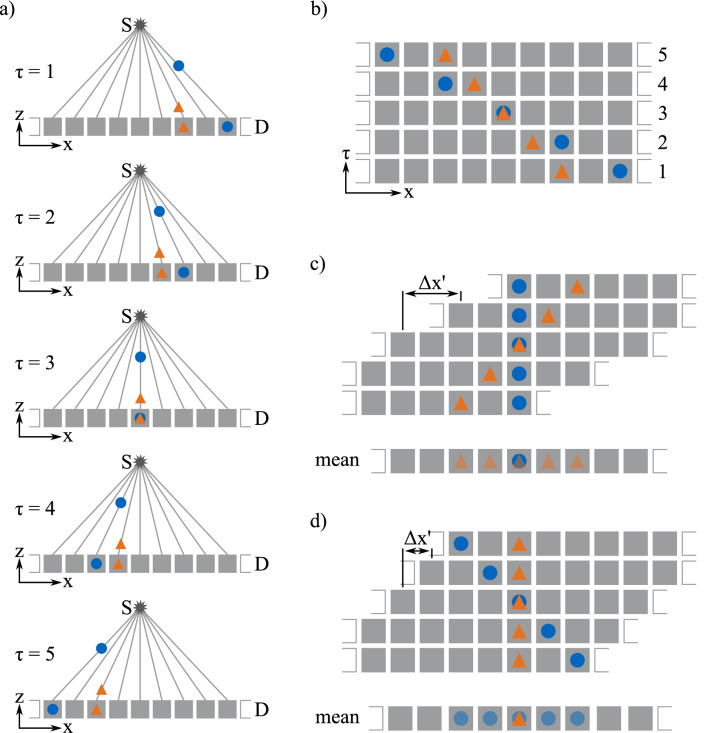
Schematic of the data resorting depicted for one modality (i.e. attenuation or dark-field). In (**a**) the path of an X-ray from the source S through a sample (blue circle above an orange triangle) is depicted for different position indices $\tau$ of the sample. On the detector D, the blue circle seemingly travels faster than the orange triangle. The resulting image stack is depicted in (**b**). The resorted data, where the blue circle is in the focal plane, is shown in (**c**). Therefore, each image had to be shifted by $\Delta x^{\prime} = \Delta x (\tau , h) / \tau$ with respect to the previous image. Taking the mean of this resorted data results in a sharp image of the blue circle and a blurred image of the orange triangle. This can be repeated for the orange triangle in the focal plane as shown in (**d**).

For image acquisition three different movements were combined. To image the sample and retrieve 3D information the sample was moved through the exposed slot of the setup. Furthermore, for the extraction of dark-field and attenuation signal a stepping of the $G_0$ grating was performed. Additionally, to improve the z-resolution the interferometer frame was rotated to image the sample at different interferometer frame positions. These three movements will be described in the following in greater detail.

The sample data was acquired by a scan-step procedure: the sample was moved linearly with a constant velocity through the exposed slot while each part of the sample received 25 X-ray pulses (pulse length 20 ms, pulse frequency 11.6 Hz) leading to a total number of 239 exposures per scan, with one scan lasting 5 s. A schematic of the different relative sample positions is given in Fig. 2a, for a sample moving from right to left. Due to the cone beam, features at different heights, depicted by a blue circle and an orange triangle, are projected onto different detector pixels, depending on the relative sample position within the exposed slot. Once the sample has been moved completely across the slot, the $G_0$ grating was moved by one stepping distance, after which another scan with 239 exposures was recorded. This was repeated seven times until the $G_0$ grating was moved over an entire period. Thus, a complete stepping curve was recorded for each pixel at every relative sample position with corresponding position index $\tau$. Flat field data is acquired by a simple phase-stepping procedure^16^. With the data from the flat-field and sample scan the mean intensity $a_f$ and interferometric visibility $V_f$ (i.e. the ratio of the amplitude and mean intensity of the stepping curve), as well as reduced intensity $a_{s,\tau }$ and reduced visibility $V_{s,\tau }$ due to the sample were calculated^18^. Subsequently the attenuation $A_\tau = -\ln (a_{s,\tau }/a_f)$ and dark-field $D_\tau = -\ln (V_{s,\tau }/V_f)$ were obtained for each position index $\tau$. This leads to a stack of 2D images with axes *x*, *y*, and $\tau$ for each modality, which is shown schematically in Fig. 2b: the upper feature, the blue circle, seems to travel faster in the recorded images than the lower feature, the orange triangle. To retrieve information about the features’ position in *z*-direction, the shift-and-add reconstruction method is used^4^. Therefore, the stack has to be rearranged. First, a specific plane in *z*-direction has to be chosen as focal plane. For this focal plane a shift $\Delta {x}$ in pixels for each $\tau$ is calculated using

1 $$\begin{aligned} \Delta {x}(\tau , h) = \frac{l_s \frac{\tau }{\tau _{max}}}{s_p \cdot \frac{{\overline{ST}}-h}{{\overline{SD}}}}, \end{aligned}$$

where $l_s$ is the scan length (i.e. how far the sample was moved during one scan), $s_p$ the pixel size of the detector, ${\overline{ST}}$ the distance between source and the table, *h* the distance between table and the focal plane, and ${\overline{SD}}$ the distance between the source and the detector. After each image in the stack has been shifted by $\Delta {x}$, only features in the focal plane are located at the same shifted *x*-position $x'$ and *y* for all $\tau$. Figure 2c,d show the shifted stacks for focal planes chosen for the two highlighted features, respectively. Averaging of the image stack in $\tau$ direction results in a sharp image of the feature in the focal plane and blurred images of features outside the focal plane. Thus, from one set of recorded data, images corresponding to different focal planes can be reconstructed. Here, images were reconstructed with focal planes from $h = {0}\,\mathrm{mm}$ to $h = {200}\,\mathrm{mm}$ in ${5}\,\mathrm{mm}$ steps. As the sample is moved only in the *x*-direction, blurring of features outside the image plane occurs only in this direction. Features parallel to the x-axis remain unblurred in y-direction for all reconstructed height slices. Furthermore, the resolution in z-direction depends on how quickly blurring increases for features which are not in the focal plane with their distance to the focal plane. This, in turn, depends strongly on the effective opening angle of the X-ray beam. As the opening angle is quite narrow in our setup, namely ${2}^\circ$, data was acquired for seven slightly overlapping positions of the interferometer frame. For each interferometer frame position the scan-step procedure, described above, was performed. This data was combined, simulating a setup with gratings covering the complete field of view of the detector. Thus, the effective opening angle of the X-ray beam was increased to ${12}^\circ$, and thus, the resolution in z-direction was improved.

### Imaging phantom

We measured the multipurpose chest phantom N1 ‘Lungman’ (Kyoto Kagaku, Kyoto, Japan) using the measurement technique described above (cf. Fig. 1b). Two 1.5 ml (diameter: 1 cm, length: 4 cm) vials (Eppendorf AG, Hamburg, Germany) filled with water and air, respectively (cf. Fig. 1c), were fixed at the inside of the phantom’s rib cage. The lung volume was filled with extruded polystyrene spheres to model the lung tissue with its many air-tissue interfaces. Please note that the bone substitutes generate a stronger dark-field signal than real bones^36^.

### Dose estimation

The dose area product was estimated from the incident air kerma, measured with a PTW NOMEX dosimeter (PTW, Freiburg, Germany) and the area of the lung. With this, the effective dose (ED) can be approximately calculated using the conversion factor given in^37^. To evaluate image quality with reduced dose, image reconstruction was repeated with subsets of the recorded data. First, the number of phase steps used for the calculation of the dark-field and attenuation images was reduced from seven to five. Then, the number of exposures per scan was reduced from 239 to 14 evenly distributed exposures. Lastly, the number of positions of the interferometer frame was decreased from seven to four, omitting every other interferometer frame position.

## Results

**Figure 3:**
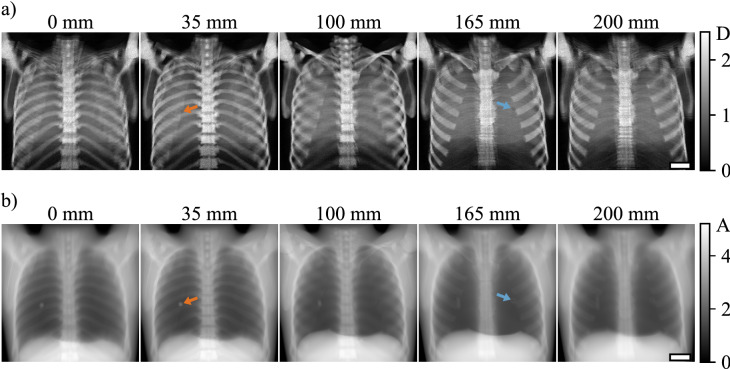
Dark-field (**a**) and attenuation (**b**) images of the phantom reconstructed for different focal planes with $h = {0}\,\mathrm{mm}$, 35 mm, 100 mm, 165 mm, and 200 mm. To simulate a grating covering the full field of view, the images were acquired for seven interferometer frame positions. Furthermore, all seven phase steps and all 239 exposures per scan were used for reconstruction. The sharpness of the bones and the vials changes for different distances between the focal plane and the sample table in both image modalities. The scale bars have a length of 5 cm.

Figure 3 shows the dark-field (a) and the attenuation (b) images of the phantom for different reconstructed heights of the focal plane. The dark-field signal of the lung is overlaid by a strong signal of the rib cage for all focal planes due to strong scattering in the bone phantom material. With changing focal planes, the sharpness of ribs changes: at $h={35}\,\mathrm{mm}$ the dorsal section of the rib cage and at $h={165}\,\mathrm{mm}$ the ventral section of the rib cage appear sharpest. In the image with $h={35}\,\mathrm{mm}$, the water-filled vial becomes apparent as a clearly delineated shadow (orange arrow in Fig. 3a). For other focal planes, this shadow is blurred or hardly visible. The air-filled vial is also visible and appears sharpest at $h={165}\,\mathrm{mm}$ (blue arrow in Fig. 3a). As the ‘Lungman’ was constructed as a thorax phantom for conventional thorax radiography and CT, the attenuation images resemble those of a real human thorax well. While the vial filled with air is better visible in the dark-field images, the contrast for the water-filled vial is stronger in the attenuation images (Fig. 3b). The vial filled with air is barely visible in any of the attenuation images. However, outline of the vial is visible. Both vials appear sharpest in the same focal planes as in the dark-field images.

**Figure 4:**
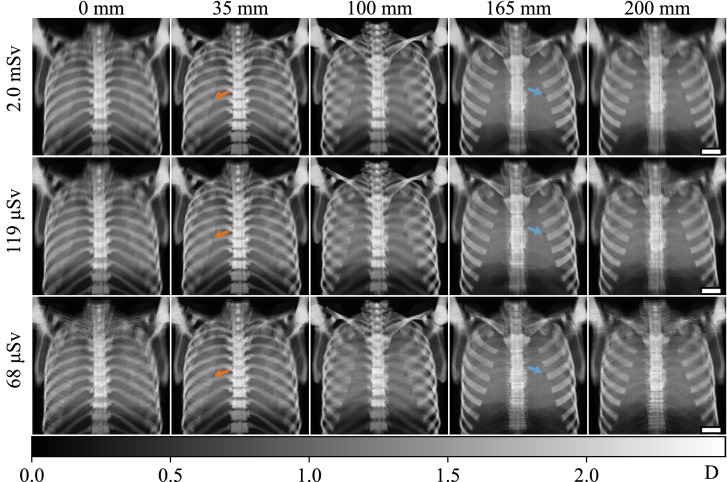
Dark-field images of the phantom for different effective doses (rows) and different focal planes (columns). The dose was reduced by successively decreasing the number of images used for reconstruction. The scale bars have a length of 5 cm. In the first row, the number of phase steps was reduced from seven to five, compared to Fig. 3. In the middle row, additionally to the reduction of phase steps from seven to five, the number of exposures per scan was reduced from 239 to 14. In the last row, the number of interferometer frame positions was decreased from seven to four, omitting every second interferometer frame position. Here, also a reduced number of phase steps (five) and exposures per scan (14) was used for reconstruction. Only in this last step, artefacts appear and affect the image quality.

For the images shown in Fig. 3 all recorded image data was used for reconstruction leading to an effective dose of 2.8 mSv. Figure 4 shows the dark-field images at reduced effective doses. In the top row, the number of phase steps was reduced from seven to five, leading to an effective dose of 2.0 mSv. Compared to the images in Fig. 3a), only the noise level in the spine changes. The blurring of features outside the focal plane remains identical. Reducing the number of exposures during one scan from 239 to 14 (middle row in Fig. 4) also only has a small impact on the image quality. However, this way the dose can be reduced significantly to 119 μSv. For the images in the bottom row, the number of interferometer frame positions was reduced, from seven to four, leading to an effective dose of 68 μSv. Ribs located outside the focal plane appear more prominent than in the previous images. However, the detectability of both inserts was not affected by the reduction in effective dose.

## Discussion

We demonstrated the retrieval of X-ray dark-field images with 3D information using a human chest phantom. By visual assessment of the dark-field images, the relative position of various features within the thorax could be determined. As both vials, filled with water and air, do not scatter X-rays, they appear as darker regions in the lung image. If the vials are not in the focal plane of the image, these spots are blurred and the difference in the dark-field signal between vial and background of the lung decreases. The contrast of the vial filled with air is higher than for the one filled with water in the dark-field images. This is likely caused by a superposition of the water-filled vial and a rib of the phantom. As real bones generate a much weaker dark-field signal, the detectability of a foreign body in the lung is expected to be less affected by their signal superposition.

While the high effective dose of 2.8 mSv in the images shown in Fig. 3 might not be a problem for non-destructive testing, it is too high for clinical imaging. Reducing the number of exposures of the sample during the image acquisition also reduces the total effective dose. However, a good image quality needs to be maintained, to prevent a reduction of the diagnostic value of this method. The dose can be lowered by reducing the number of the phase steps. Still, enough steps are needed in order to calculate the different attenuation and dark-field images. Reducing the number of exposures during one scan or the number of interferometer frame positions, reduces the number of angles under which a voxel is sampled. If the number of angles is too small or not evenly distributed, artefacts can arise in the final images. The artefacts appear as multiple images of the features, as can be seen for the clavicles in the image taken at $h={0}\,\mathrm{mm}$ with 68 μSv. The clavicles are far from the image plane and appear several times. From this observation, it is clear that the angular sampling is insufficient to smear them out properly. Nevertheless, both vials are still visible in these images. At the same time the effective dose applied is only two-times higher than for clinical X-ray dark-field radiography^34^.

The results presented here are a first proof of principle. To determine the diagnostic value of this method in a clinical application, an extensive study would have to be performed. Moreover, the best trade-off between image quality and dose has to be determined. The larger the angle range, i.e. total beam divergence, the better is the z-resolution. The beam divergence of the current setup is insufficient to achieve a reasonable z-resolution with a set of data from one single interferometer frame position. This problem was here circumvented by recording seven data sets with different interferometer frame positions. Consequently, the measurement procedure is time consuming. This might not be a problem in material testing, but it is not practical for clinical imaging. Additionally, the fabrication of large gratings covering the complete area of the detector has not yet been achieved. Constructing a setup with multiple slot interferometers with different projection angles could be a solution to this problem. With such a setup, acquisition of attenuation and dark-field images at a relatively low effective dose might be possible. In our study, the dose was also reduced by decimating the acquired exposures per scan. In future studies this could be achieved by moving the sample faster trough the exposed slot while the X-ray pulse frequency and exposure time are the same as in the high dose acquisition. However, this might lead to motion artefacts. Another possibility would be to use the same velocity of the sample and exposure time as in the high dose acquisition but reduce the X-ray pulse frequency. The reconstruction method used here was a simple shift-and-add method. This leads to several appearances of features if the angular sampling is insufficient. However, there are other reconstruction methods like filtered back projection or iterative reconstruction, which might be able to reduce those artefacts. Furthermore, as seen with the vial filled with water, a superposition of the signal from the examined feature with the signal from other objects could hamper the detectability of the feature. Further studies should be conducted to evaluate the magnitude of this effect for different types of samples. A dark-field CT does not suffer from the superposition of structures. However, combining dark-field with large field of view CT is technically challenging. Therefore, the method presented here offers a good trade-off: it supplies more information than X-ray dark-field radiography, but it is technical easier to implement than a large field of view dark-field CT.

In conclusion, we were able to gain 3D information of the position of features in the sample in X-ray dark-field imaging. However, the presented study is only a first proof of principle and the benefits from this method for non-destructive testing and clinical applications has to be demonstrated in further studies.
